# MicroRNA-375 targets the 3-phosphoinositide-dependent protein kinase-1 gene in pancreatic carcinoma

**DOI:** 10.3892/ol.2013.1510

**Published:** 2013-08-02

**Authors:** SHI-DUO SONG, JIAN ZHOU, JIN ZHOU, HUA ZHAO, JIAN-NONG CEN, DE-CHUN LI

**Affiliations:** 1 Department of General Surgery, The First Affiliated Hospital of Soochow University, Jiangsu 215006, P.R. China; 2 Jiangsu Institute of Hematology, Soochow University, Suzhou, Jiangsu 215006, P.R. China

**Keywords:** microRNA, microRNA-375, pancreatic carcinoma, 3-phosphoinositide-dependent protein kinase-1

## Abstract

Pancreatic carcinoma (PC) is an aggressive malignancy with one of the poorest mortality rates. It is the sixth leading cause of mortality from malignant disease in China and the fourth leading cause of cancer-related mortality in the United States. The poor outcome reflects the requirement for an improved understanding of the transcriptional control of oncogenic signaling pathways. 3-phosphoinositide-dependent protein kinase-1 (PDK1) is a potent oncogenic driver of PC. The present study aimed to elucidate the transcriptional regulation of microRNA (miR)-375-targeted PDK1. miR-375 is a putative target and, in the present study, was observed to be significantly downregulated in the tumor compared with non-tumor tissues from patients with PC (n=44). As determined by a luciferase reporter assay, the ectopic expression of miR-375 was identified to diminish the transcriptional activity of PDK1. Furthermore, immunoblotting revealed that miR-375 suppressed endogenous PDK1 protein levels. Functional assays showed that miR-375 was able to inhibit proliferation and promote apoptosis of the PC cells. miR-375 is a significant regulator of the PDK1 oncogene, suggesting that it may have a potential therapeutic role in the treatment of PC.

## Introduction

Pancreatic carcinoma (PC) is an aggressive malignancy with one of the poorest mortality rates. It is the sixth leading cause of mortality from malignant disease in China and the fourth leading cause of cancer-related mortality in the United States (1–3). The estimated mortality is almost equal to the estimated incidence, with an overall 5-year survival rate of <5% (3). Therefore, the identification of new associated factors and novel therapeutic targets for PC remains an imperative clinical issue. K-ras, CDKN2A, p53, DPC4 and 3-phosphoinositide-dependent protein kinase-1 (PDK1) are known to be mutated or inactivated during PC tumorigenesis, and mediate other functional and relevant cancer pathways (4–7). PDK1 is a key component in phosphatidylinositol 3-kinase-Akt-mammalian target of rapamycin (PI3K-Akt-mTOR) signaling, a well-documented pathway that regulates cancer cell survival and proliferation (7). PDK1 is a pleckstrin homology (PH) domain-containing protein that is activated following PI3K activity, which in turn phosphorylates Akt1 at threonine 308 (or cognate locations on other isoforms) along with a large variety of other AGC kinase substrates. Although this kinase has a significant role in PI3K-Akt-mTOR signaling, the activating mutations of the gene encoding PDK1 have not been described. The deregulation of PDK1 is critical to the progression of pancreatic tumors.

MicroRNAs (miRNAs/miRs) are non-coding RNAs that consist of 21–23 nucleotides and are a recently emerging class of endogenous negative regulators of gene expression that possess a remarkable evolutionary conservation (8,9). miRNAs are believed to modulate gene expression at the post-transcriptional level (10,11). These small molecules exert their regulatory effects by base-pairing to partially complementary mRNA and functioning by two mechanisms, the degradation of target mRNA transcripts or the inhibition of mRNA translation (11,12). miRNAs are also associated with the main phenotypes of numerous cancer cells (including PC), such as proliferation, invasion and apoptosis (13–15). Therefore, studying the functions and mechanisms of miRNAs may lead to new approaches for the categorization, diagnosis and treatment of human cancers. miR-375 is differentially expressed in various neoplasms (16–18). The effects of miR-375 may be cell-type specific (16). Few studies of PC have focused on targeting the clinical and prognostic significance of miR-375.

Our previous study identified that miR-375 is involved in PC through the suppression of PDK1 (19). However, the detail of how miR-375 regulates PDK1 expression in PC remains unknown.

The present study suggests that miRNAs may play key roles in tumorigenesis by regulating the expression of genes that are associated with oncogenic signaling pathways. However, further studies are required in order to explore and validate whether the putative target, miR-375, is involved in the regulation of the PDK1 oncogene.

## Materials and methods

### Tissue samples

PC tissues and their respective adjacent normal tissues were obtained post-operatively from 44 patients in 2009 from the Department of General Surgery, The First Affiliated Hospital of Soochow University (Suzhou, Jiangsu, China). Informed consent was obtained from all patients for their tissues to be used for scientific research. Approval for the study was obtained from the Department of General Surgery, The First Affiliated Hospital of Soochow University. All diagnoses were based on pathological and/or cytological evidence. The histological features of the specimens were evaluated by two senior pathologists according to the classification criteria from the World Health Organization (20). The tissues were obtained from the patients prior to chemotherapy or radiation therapy. The specimens were immediately frozen and stored at −80°C prior to the microarray and quantitative (q)PCR analyses.

### Cell culture

The human PC cell line, Panc-1, was maintained in DMEM supplemented with 10% FBS. The cells were cultured in an incubator at 37°C with 5% CO_2_.

### qPCR

Total RNA was extracted from the patients or the cell line samples using TRIzol (Invitrogen, Carlsbad, CA, USA), according to the manufacturer’s instructions. The miR-375 expression level was determined by qPCR using Taqman assay kits (Applied Biosystems, Foster City, CA, USA), with U6 small nuclear RNA as an internal normalized reference. For the quantification of PDK1 and β-actin, the extracted RNA was reverse transcribed to cDNA using an oligo(dT) 12 primer and Superscript II (Invitrogen). The primers for these genes are summarized in Table I. The relative expression levels of the primers were measured in triplicate on a Prism 7900 Real-Time PCR machine (Applied Biosystems), according to the manufacturer’s instructions.

### Vector construction

To generate a miR-375 expression vector (pcDNA 3.1-miR-375) containing a miR-375 precursor, a sequence was amplified using the following primers: Forward, 5′-CCGCTCGAGCAGATGCGTTCAGGTGAG-3′ and reverse, 5′-CGGAATTCTGGCGGCGGCAGGTGCCTG-3′. The amplified region was cloned into the pcDNA 3.1^+^ vector (Genechem, Shanghai, China) using *Xba*I and *Hin*dIII restriction sites and confirmed using DNA sequencing, as previously described (21).

The putative miR-375 binding site at the 3′ UTR of PDK1 was cloned downstream of a CMV promoter-driven firefly luciferase cassette in a pcDNA 3.1 vector (Genechem). A mutant form of this luciferase construct was also generated using the PCR-based overlap-extension procedures, as reported previously (22).

### Transfection

Chemically synthesized RNAs, including scramble, an miR-375 mimic and its inhibitor, were obtained from GenePharma (Shanghai, China). For transfection, the cells were grown on 24-well culture plates to 70–80% confluence. Following 24 h, the cells were cotransfected with Renilla luciferase reporter (PRL; 0.1 μg), the previously constructed reporter plasmids (0.5 μg) and chemically synthesized RNA (0.5 μg) using Lipofectamine 2000 transfection reagent (Invitrogen). Subsequent to being incubated for 24 h, the cells were harvested using lysis buffer for use in the luciferase assay. G418 was then used to screen the monoclonal cells.

### Luciferase reporter assay

For the luciferase reporter assay, Panc-1 cells (3×10^4^) were plated in a 24-well plate and then cotransfected with 400 ng pcDNA 3.1-miR-375 or pcDNA 3.1 empty vector, 200 ng wild-type or mutant luciferase construct and 40 ng PRL-TK (Promega, Madison, WI, USA) using Lipofectamine 2000 (Invitrogen) according to manufacturer’s instructions. The cells were collected at 48 h post-transfection and analyzed using the Dual-Luciferase Reporter Assay System (Promega, Fitchburg, WI, USA). The pRL-TK vector provided the constitutive expression of Renilla luciferase and was used as an internal control to correct the differences in the transfection and harvest efficiencies (23). Each treatment was performed in triplicate and repeated in three independent experiments.

### Western blotting

The treated cells were washed twice with ice-cold PBS, then directly lysed with 200 μl 2× SDS cell lysis buffer in each well of a 6-well plate cluster. The lysates were boiled, centrifuged at 15,680 × g and loaded onto a 12% SDS-PAGE gel. The samples were electrophoresed for 4 h and transferred to a Millipore Immobilon transfer membrane (Millipore, Billerica, MA, USA) in Bio-Rad blot apparatus (Bio-Rad, Hercules, CA, USA). Subsequent to being blocked with 5% skimmed milk in PBS-Tween-20 for 1 h at room temperature, the membranes were blotted with a goat polyclonal anti-hPDK1 primary antibody overnight at 4°C. The membranes were washed with TBST prior to being incubated with mouse polyclonal anti-goat secondary antibody linked to horseradish peroxidase (dilution, 1:2000) for 1 h at room temperature. The membranes were washed again with TBST and the blot was incubated in a detection reagent (ECL Advance Western blotting detection kit; Amersham Bio-science, Freiburg, Germany) and exposed to a Hyperfilm ECL film (Pierce, Rockford, IL, USA). Anti-PDK1 (Santa Cruz Biotechnology Inc., Santa Cruz, CA, USA) and GAPDH (KangChen Bio-Tech, Shanghai, China) antibodies were used in the western blot analyses.

### Cell proliferation

The cells were seeded in a 96-well plate at a density of 10,000 cells per well and incubated for 96 h. The *in vitro* growth was measured using Cell Counting kit-8 (CCK-8; Dojindo Laboratories, Kumamoto, Japan). The optical density (OD) at 450 nm was measured using a Microplate Reader (Bio-Rad) and the proliferation index was calculated as the experimental OD value/control OD value. A total of three independent experiments were performed in quadruplicate.

### Cell cycle analysis

The cells were washed three times using cold PBS and then fixed in 70% ethanol in PBS at −20°C for 2 h. Following fixation, the cells were washed with cold PBS and stained with 1 ml propidium iodide (PI) staining buffer (MultiScience, Hangzhou, China), which contained 200 mg/ml RNase A and 50 mg/ml PI, at room temperature for 30 min in the dark. The analyses were performed on a FACScan flow cytometer (Becton-Dickinson, Sunnyvale, CA, USA). The experiments were repeated three times.

### Cell apoptosis

The quantification of the apoptotic cells was performed using the Annexin-V-FITC Apoptosis Detection kit (Invitrogen), according to the manufacturer’s instructions. Early apoptotic cells were defined as Annexin-V-positive, PI-negative cells. The analyses were performed on a FACScan flow cytometer (Becton-Dickinson). The experiments were repeated three times.

### Tumorigenicity assays in nude mice

Six-week-old female BALB/c athymic nude mice were subcutaneously injected into the right armpit region using 1.5×10^6^ cells in 0.15 ml PBS. The following groups of mice (n=5 per group) were tested: Group I (miR-375 mimics) was injected with Panc-1 cells transfected with miR-375 mimics; group II (Mock) was injected with Panc-1 cells transfected with negative control; and group III (Vec) was injected with Panc-1 cells alone. The tumor size was measured every 2 or 3 days using calipers. The tumor volume was calculated with the following formula: (L × W^2^)/2, where L is the length and W is the width of the tumor. The mice were sacrificed at four weeks and the weights of the tumors were measured. All experimental procedures involving animals were in accordance with the Guide for the Care and Use of Laboratory Animals (NIH publication no. 80–23, revised 1996) and were performed according to the ethical guidelines for animal experiments at the Department of Clinical Immunology, Soochow University (Suzhou China).

### Statistics

The differences between the groups were evaluated using the Student’s t-test. P<0.05 was considered to indicate a statistically significant difference.

## Results

### miR-375 is a candidate miRNA that regulates the expression of PDK1

**Figure 1:**
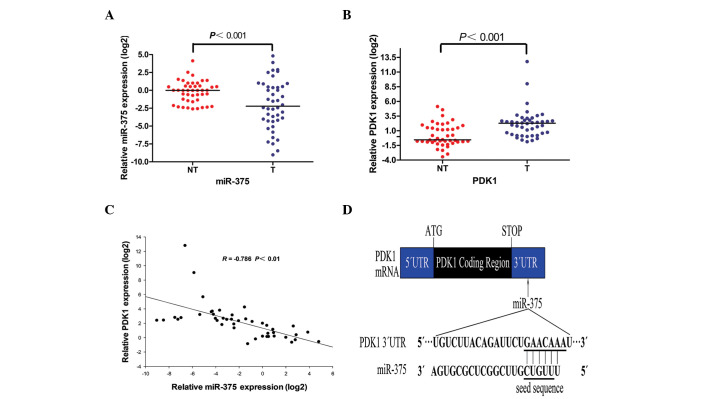
miR-375 is a candidate miRNA that regulates PDK1. (A) qPCR analysis showing the downregulation of miR-375 mRNA in the PC patients. (B) qPCR analysis showing the upregulation of PDK1 mRNA in the PC patients. (C) An inverse correlation of PDK1 and miR-375 expression levels was examined using a Spearman correlation analysis. (D) Schematic of the *in silico* analysis of the predicted binding sites showing that miR-375 binds to the 3′ UTR region of PDK1 mRNA. The predicted consequential pairing between the target region (position 3490–3496 of PDK1 3′ UTR) and the seed sequence of miR-375 is shown. PDK1, 3-phosphoinositide-dependent protein kinase-1; qPCR, quantitative PCR; miR-375, microRNA-375, PC, pancreatic carcinoma; NT, non-tumor; T, tumor.

Bioinformatic algorithms (TargetScan 4.2; Whitehead Institute for Biomedical Research, Cambridge, MA, USA) were used to identify the candidate miRNAs that targeted the 3′ UTR region of the PDK1 gene, and to subsequently identify miR-375 for further studies (Fig. 1D).

A qPCR analysis of miR-375 and PDK1 was performed in 44 pairs of PC tumor and matched adjacent non-tumor tissues. The results show that miR-375 was significantly down-regulated in the PC tumor tissues (Fig. 1A), while PDK1 was upregulated in the PC tumor tissues (Fig. 1B). PDK1 was inversely correlated with miR-375 (Fig. 1C), suggesting that PDK1 is a target of miR-375.

### PDK1 is a direct target of miR-375

**Figure 2:**
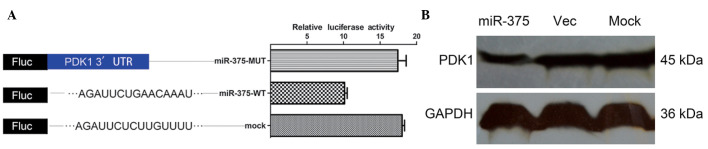
PDK1 is a direct target of miR-375. (A) PC cells were cotransfected with Renilla luciferase control and firefly luciferase reporter containing either wild-type (WT) or seed region mutated (MUT) PDK1 3′ UTR and/or the pcDNA3.1-miR-375 (miR-375). (B) The cells were transfected with miR-375 or the pcDNA3.1 empty vector (Vec), and western blotting was used to show the suppressed expression of endogenous PDK1 in Panc-1 cells following miR-375 transfection (P<0.05). PDK1, 3-phosphoinositide-dependent protein kinase-1; miR-375, microRNA-375; PC, pancreatic carcinoma, Fluc, firefly luciferase.

To confirm that PDK1 is a target of miR-375, a luciferase reporter assay was performed. The relative luciferase activity of the reporter that contained the wild-type 3′ UTR was significantly suppressed when miR-375 was cotransfected (Fig. 2A). In contrast, the luciferase activity of the mutant reporter was unaffected by the cotransfection of miR-375 (Fig. 2A), indicating that miR-375 suppressed gene expression using the miR-375-binding sequence at the 3′ UTR of the PDK1 gene.

The effect of miR-375 on the endogenous expression of PDK1 was further examined. The ectopic expression of miR-375 caused a decrease in PDK1 protein levels in the Panc-1 cells (Fig. 2B).

### miR-375 suppresses PC cell proliferation

The significant reduction of miR-375 in the PC samples and its inhibitory effect on the PDK1 oncogene prompted an investigation into its possible biological role in cancer cells. To study the effect of miR-375 on cell proliferation, a CCK-8 proliferation assay was performed.

**Figure 3:**
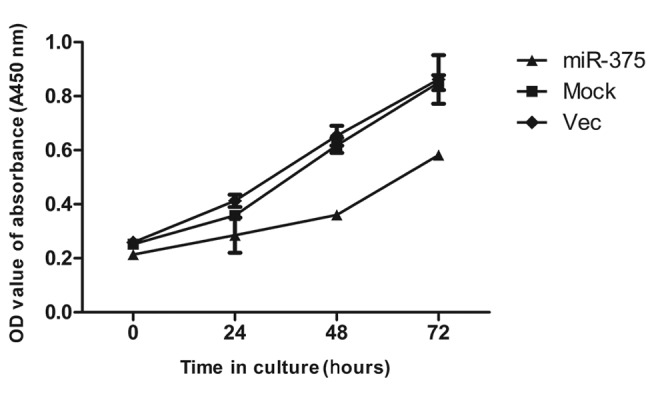
Association of miR-375 with the proliferation activities of PC cells. Ectopic expression of miR-375 was achieved by transfecting pcDNA3.1-miR-375. PC cells without any treatment (mock) and those that were transfected with pcDNA3.1 empty vector (Vec) were used as controls. A CCK-8 assay was performed to determine the cell proliferation activity. The data shown are the representative set of three independent experiments, and are expressed as mean ± SEM. P<0.05, relative to the mock control. miR-375, microRNA-375; PC, pancreatic carcinoma; OD, optical density; CCK-8, cell counting kit-8.

The results showed that the proliferation of the pcDNA 3.1-miR-375-transfected cells was slower than the mock and empty vector-treated cells (Fig. 3).

### miR-375 arrests the cell cycle at G_0_/G_1_ and induces PC cell apoptosis

**Figure 4:**
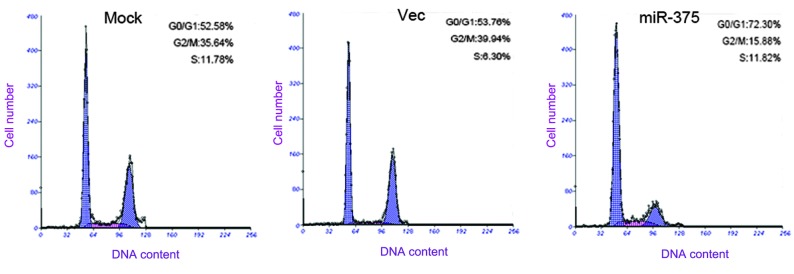
Association of miR-375 with the cell cycle of the PC cells. FCM was performed to determine the cell cycle. The data show that the PC cells that were transfected with miR-375 underwent a cell cycle arrest at the G_0_/G_1_ phase (P<0.05). PC, pancreatic carcinoma; FCM, flow cytometry; miR-375, microRNA-375.

To determine the effect of miR-375 on apoptosis and the cell cycle of the PC cells, flow cytometry (FCM) was performed. The results reveal that the Panc-1 cells that were transfected with the miR-375 mimics had an evident cell cycle arrest at the G_0_/G_1_ phase in contrast with the mock and empty vector-treated cells (Fig. 4).

**Figure 5:**
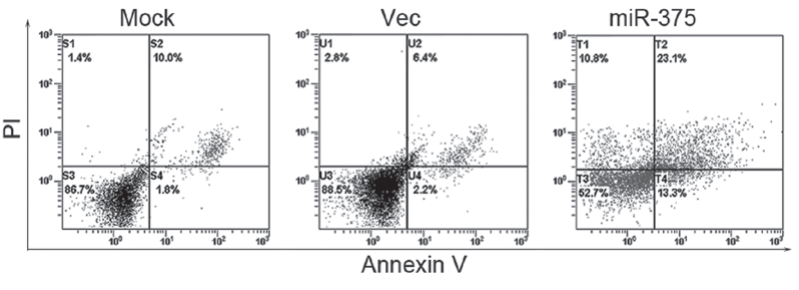
Association of miR-375 with the apoptosis of the PC cells. FCM was performed to determine cell apoptosis. The data show that the apoptotic activities of the pcDNA3.1-miR-375-transfected cells were higher than that of the mock and empty vector-treated cells (P<0.05). miR-375, microRNA-375; PC, pancreatic carcinoma; FCM, flow cytometry; PI, propidium iodide.

The results show that the apoptotic activities of the pcDNA 3.1-miR-375-transfected cells were higher than that of the mock and empty vector-treated cells (Fig. 5).

### miR-375 suppresses tumorigenicity in vivo

**Figure 6:**
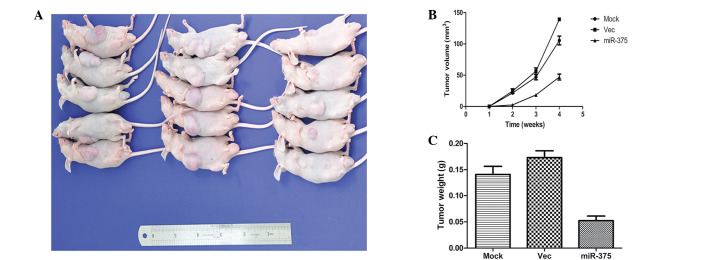
miR-375 suppresses tumorigenicity *in vivo*. Three groups of mice (n=5 per group) were tested. (A) The size of the tumors in three groups. (B) The tumor growth curves of the three groups for four weeks. (C) The mean tumor weight of the three groups at the end of the experiment. Data are presented as mean ± standard deviation (P<0.05). miR-375, microRNA-375.

To confirm the aforementioned findings, an *in vivo* tumor model was used. miR-375 mimic-transfected Panc-1 cells (miR-375 mimics), negative control-transfected Panc-1 cells (mock) and Panc-1 cells were injected separately into three groups of nude mice (n=5 per group). At four weeks post-injection, the miR-375 mimics group had developed substantially smaller tumors than the other two groups (Fig. 6A). The tumor volume at the time of death of the mice that were injected with miR-375 mimic-transfected cells was 60.49±4.59 mm^3^, whereas the tumor volumes of the mice injected with mock or Panc-1 cells were 139.28±3.21 mm^3^ and 105.47±5.89 mm^3^, respectively (Fig. 6B). Furthermore, the mean tumor weight at the end of the experiment was markedly lower in the miR-375 mimics group (0.052±0.013 g) compared with the mock and Panc-1 groups (0.173±0.028 g and 0.141±0.031 g, respectively; Fig. 6C).

## Discussion

The present study identified that miR-375 was expressed at a significantly lower level in PC tissues than in the respective adjacent normal tissues. In the human clinical specimens, the miR-375 mRNA levels were inversely correlated with those of PDK1. In addition, miR-375 directly regulated the expression of PDK1. The PDK1 oncogene was identified to be negatively regulated by miR-375 at the post-transcriptional level through a specific target site within the 3′ UTR of the gene. The overexpression of miR-375 may have resulted in the downregulation of PDK1. Genomic amplification of PDK1 and the loss of miR-375 may result in the enhanced expression of the PDK1 oncogene and, in turn, promote PC development, including the inhibition of cell proliferation, the induction of apoptosis and cell cycle arrest at G_0_/G_1_. PDK1 is a potent regulator in cell growth and a previous study supports its oncogenic role in mammalian cells (7).

Numerous growth factors activate the PI3K pathway, which in turn phosphorylates phosphatidylinositol-4,5-biphosphate (PIP2) to generate phosphatidylinositol-3,4,5-triphosphate (PIP3). An extensively studied signaling event controlled by PIP3 is the activation of a group of AGC family protein kinases, including isoforms of protein kinase B (Pkb/Akt) and the ribosomal S6 kinase (S6K), which play crucial roles in regulating the physiological processes that are relevant to metabolism, cellular growth, proliferation and survival (24,25). PDK1 is a PH domain-containing protein that is activated following PI3K activity, which in turn phosphorylates Akt1 at threonine 308 (or cognate locations on other isoforms), along with a large variety of other AGC kinase substrates. Although this kinase has a significant role in PI3K-Akt-mTOR signaling, the presence of activating mutations of the gene encoding PDK-1 have not been described. A study by Westmoreland *et al*(7) suggested that PDK-1 expression levels may control the proliferation, survival and growth of developing pancreatic cells in PC, but this hypothesis has not been fully tested. PDK1 activation is primarily dependent on cytoplasmic membrane localization, and is considered to be constitutively active. Thus, while it is unlikely that there are activating mutations in the kinase domain, it is possible that membrane-targeting PDK1 mutations may result in pathway activation (26).

PDK1 is a gene that has been identified as a direct target of miR-375 (27) and is a key component in Akt signaling, a well-documented pathway that regulates cancer cell survival and proliferation.

The overexpression of miR-375 in PC cells may reduce cell proliferation and induce cell apoptosis, suggesting a fundamental role for miR-375 in the development of PC. Accumulating studies have revealed the significance of miRNAs in regulating the growth and apoptosis of cancer cells. miR-21 and -155 have been suggested to function as proto-oncogenes and have been shown to be overexpressed in several cancers (28). miRNA-146a, which is downregulated in various cancer types, has been shown to regulate cell proliferation and apoptosis (29).

A reduction in the expression of miR-375 has been reported in PC (30), hepatocellular carcinoma (HCC) (18) and head and neck squamous cell carcinoma (17). With the exception of its role in cancer, miR-375 is also a significant regulator in mammalian pancreatic islet-cell development and in the regulation of insulin secretion (31), thus indicating its diverse role in normal physiology. The target genes of miR-375 may function cooperatively through different cellular mechanisms. The identification of PDK1 as a target of miR-375 provides new insights into the molecular networks of miR-375. Further studies are required to investigate the targets of miR-375 that may favor the process of tumorigenesis. An introduction of a single miRNA may be beneficial in modulating the complex downstream signals and halting the process of tumorigenesis.

To date, yes-associated protein (YAP) (32), JAK2 (33) and 14-3-3ζ (27) are other genes that have been identified as direct targets of miR-375. PDK1 has also been identified as a target of miR-375 in esophageal (34) and gastric cancer (35).

The present study identified that the ectopic expression of miR-375 may suppress PDK1 mRNA and protein levels, indicating that miR-375 may also interfere with PI3K-Akt-mTOR signaling. Therefore, miR-375 is a potential therapeutic target against the PI3K-Akt-mTOR signaling axis for preventing PC development and progression.

## Figures and Tables

**Table I:** Primer sequences for qPCR analysis.

Gene	Forward primer (5′→3′)	Reverse primer (5′→3′)
PDK1	GTGTAGATTAGAGGGATG	AAGGAATAGTGGGTTAGG
β-actin	CTCCATCCTGGTCTCGCTGT	GCTGTCACCTTCACCGTTCC

PDK1, 3-phosphoinositide-dependent protein kinase 1; qPCR, quantitative PCR.
